# Systemic biochemical changes in pepper (*Capsicum annuum* L.) against *Rhizoctonia solani* by kale (*Brassica oleracea* var. *acephala* L.) green manure application

**DOI:** 10.1186/s12870-023-04525-z

**Published:** 2023-10-26

**Authors:** Víctor M. Rodríguez, Pablo Velasco, María Elena Cartea, Jorge Poveda

**Affiliations:** 1 Group of Genetics, Breeding and Biochemistry of Brassicas. Mision Biologica de Galicia (MBG-CSIC), Pontevedra, 36143 Spain; 2 Recognized Research Group AGROBIOTECH, Consolidated Research Unit 370 (JCyL), Department of Plant Production and Forest Resources, Higher Technical School of Agricultural Engineering of Palencia, University Institute for Research in Sustainable Forest Management (iuFOR), University of Valladolid, Avda. Madrid 57, Palencia, 34004 Spain

**Keywords:** Glucosinolates, Salicylic acid, Ethylene, Glucobrassicin, Elicitors

## Abstract

**Background:**

In the search for new alternatives to avoid the problems associated with the use of synthetic chemical fungicides in agriculture, the use of green manure (GrM) could help combat fungal diseases of crops, such as those produced by the necrotrophic pathogen *Rhizoctonia solani*. In the case of the use of *Brassica* tissues as GrM, it could have an elicitor capacity for systemic plant resistance.

**Results:**

We used kale leaves as a GrM and applied it to pepper plants infected with *R*. *solani*. The application of freeze-dried kale tissues to the roots of pepper plants produced a systemic activation of foliar defences via the salicylic acid (SA) and ethylene (ET) pathways, significantly reducing pathogen damage. In addition, this systemic response led to the accumulation of secondary defence metabolites, such as pipecolic acid, hydroxycoumarin and gluconic acid, in leaves. Remarkably, pepper plants treated with lyophilised kale GrM accumulated glucosinolates when infected with *R*. *solani*. We also confirmed that autoclaving removed part of the glucobrassicin (85%) and sinigrin (19%) content of the kale tissues.

**Conclusions:**

GrM kale tissues can activate systemic defences in bell pepper against foliar pathogens through SA/ET hormonal pathways, accumulating secondary defence metabolites.

## Introduction

Today, agricultural systems are facing the great challenge of feeding a growing world population. The increase in agricultural productivity over the last few decades relies on the massive use of agrochemicals (fertilisers and pesticides) [1]. However, the use of synthetic chemical pesticides has led to the accumulation of toxic residues in soil, water and food, causing serious environmental (loss of biodiversity, destruction of ecosystems) and human health problems (from topical irritations to serious immunological and hormonal damage, and even cancer) [2, 3]. Synthetic chemical fungicides are the largest group of pesticides used globally, accounting for more than 30% of the total market, and there has been a continuous reduction in the efficacy of these compounds as a result of the development of antifungal resistance by agricultural plant pathogenic fungi [4]. Therefore, current and future agriculture needs to develop new effective, healthy and environmentally friendly strategies for the control of plant pathogenic fungi, with microbial pesticides and phytochemical-based pesticides being the most developed and studied strategies [5].

Green manure (GrM) is a widespread agricultural technique in organic farming based on the burying of cover crops for incorporation into the soil. This increases the soil organic C and N content, aggregate stability, microbial biomass, and enzyme activity in the soil [6]. Some of the main crops used as GrM are species within the *Brassica* genus, especially for leguminous crops [7], being an important source of nutrients, such as N and S [8]. In addition, *Brassica* GrM acts directly against plant pathogens in the soil due to its content of antimicrobial phytochemicals [9]. In pepper, soil incorporation of *Brassica* GrM significantly delays *Phytophthora capsica* disease incidence progression by 83% [10]. Among crops belonging to the *Brassica* genus, kale (*Brassica oleracea* var. *acephala*) has gained popularity in recent years among consumers as a “superfood” due to its nutraceutical and therapeutic potential [11]. This is due to the presence of different bioactive phytochemicals, such as pigments, carotenoids, polyphenols and glucosinolates (GSLs), in its tissues, as well as its antioxidant activity [12–14]. GSLs stand out, being mainly indole GSLs [glucobrassicin (3-indolylmethyl GSL) and 4-hydroxyglucobrassicin (4-hydroxy-3-indolylmethyl GSL)] and aliphatic GSLs [sinigrin (2-propenyl GSL) and progoitrin (2-(R)-2-hydroxy-3-butenyl GLS)] [15, 16]. GSLs are sulphur-rich secondary metabolites, namely β-thioglucoside N-hydroxysulphates, involved in numerous physiological processes in plants within the order Brassicales [17]. One of their main functions is as defence metabolites against pests, as they are hydrolysed by myrosinase enzymes to highly toxic compounds, such as isothiocyanates [18]. In addition, both GSLs and their GSL hydrolysis products (GHPs) are potent antimicrobials, effectively used in the control of plant-parasitic nematodes [19, 20], fungi and oomycetes pathogenic to crops [21, 22].

*Rhizoctonia solani* is a widely distributed, necrotrophic, soil-borne pathogen that severely affects a wide diversity of economically important crops [23]. In its host plants, *R*. *solani* causes diverse symptoms, including pre- and post-emergence damping-off, seed, root, hypocotyl, crown, stem, branch or pod rot, seed blight, black scurf, or stem canker [23]. In adult plants, *R*. *solani* symptoms can be observed mainly on roots and stems but also on leaves [24]. This fungus penetrates the leaf intercellular spaces through natural openings, such as stomata, and can infect crops, such as rice [25, 26], turnip green [27], oil palm [28], and cassava [29]. In pepper, the main symptoms caused by *R*. *solani* are seedling damping-off, root and stem rot, and necrotic spots on the hypocotyl [30, 31].

To the best of our knowledge, no studies have been carried out on how GrM from *Brassica* crops activates the systemic defences of other crops. In this sense, the main objective of this work is to use kale GrM to induce systemic acquired resistance (SAR) in peppers against *R*. *solani* foliar infection by analysing the hormone and metabolomic pathways involved.

## Material and methods

### Organisms used

Bell pepper plants (*C*. *annum*) from the Protected Geographical Indication (PGI) "Pemento de Mougán" (Galicia, Spain) were used in this study. GrM tissues were obtained from kale plants grown in the field without any fertilisation or phytosanitary treatment. Bell pepper and kale seeds were obtained from the germplasm bank of the Misión Biológica de Galicia (MBG-CSIC) (Pontevedra, Spain) under the identifications MBG-P001F29 and MBG-BRS0062, respectively.

The plant pathogen fungus *R*. *solani* (CRD 207/99 JCYL 957), anastomosis group three, isolated from a potato crop, was provided by the Regional Diagnostic Center of the Regional Government of Castilla y León (Salamanca, Spain). The fungus was grown on potato dextrose agar (PDA) medium (Sigma-Aldrich, St. Louis, MO, USA).

### Bell pepper growth and kale tissue application

Pepper seeds were surface sterilised by vigorous sequential shaking in 70% ethanol and 5% sodium hypochlorite solutions for 10 min each and then washed thoroughly four times in sterile distilled water. Pepper seeds were individually transferred to 0.2 L pots containing a substrate consisting of peat moss (Profi-Substract, Gramoflor, Valencia, Spain) previously sterilised in an autoclave (twice, 24 h apart) and maintained in a greenhouse. Plants were watered 2–3 times per week, according to the observed needs, always with the same amount of water in all plants. No exogenous fertilisation was used. Greenhouse conditions were as follows: 14 h photoperiod, ambient temperature (12–30 °C), and relative humidity above 80%.

Kale leaves were used as GrM. From 8 kale plants, 3 random leaves were collected in the field from each plant when they were fully developed (14-week-old plants). Of the total pool of leaves (24), half were frozen in liquid nitrogen and stored at − 80 °C. We used autoclaved leaves (120 °C, 20 min) as a control to determine whether the metabolites present in the kale GrM were responsible for the putative beneficial effect. Subsequently, both leaf pools were freeze-dried in a lyophiliser (GAMMA 2–16 LSC plus, Christ, Germany) and mechanically milled to a fine powder in a grinder (Janke and Kunkel A10 mill, IKA-Labortechnik, Staufen, Germany).

The application of kale tissues was carried out in six-week-old pepper seedlings when they had 5–6 true leaves. The bell pepper plants were transplanted into 5 L pots containing a substrate consisting of peat moss (Profi-Substract, Gramoflor, Valencia, Spain), previously sterilised in an autoclave (twice, 24 h apart). In each of the holes where the pepper roots were introduced, 1 g of different kale leaf powders (GrM) was applied. Thus, the final experiment setup consisted of three treatments: (1) plants without GrM used as a control (C), (2) plants inoculated with freeze-dried GrM (FD-GrM), and (3) plants inoculated with autoclaved and freeze-dried GrM (AUT-GrM). A total of 20 plants (in 20 pots) were used for each treatment.

### *Rhizoctonia solani* infection and lesion analysis

The fungal plant pathogen *R*. *solani* was used to infect the bell pepper leaves. Agar plugs of 6 mm were obtained from Petri dishes where the fungus was actively growing (edges of colonies). The mycelium of the plugs was placed in contact with bell pepper leaves one week after transplanting by depositing 5 µl of sterile agarose on the leaf surface as “a glue” for the fungal plug. Inoculation was made in the medium part of the third true leaf. Each leaf was covered individually with a plastic bag to maintain a high relative humidity. Leaves were collected for analysis one week after *R*. *solani* infection. A total of 10 plants were infected for each treatment. The area of the lesion produced on each leaf was quantified using ImageJ software (US National Institutes of Health, Bethesda, U.S.A.). The entire experiment was replicated three times.

### GSL analysis

Leaf GSL analysis used as GrM (*B*. *oleracea*-autoclaved and *B*. *oleracea*) was performed following the methodology described by Velasco et al. [32]. The analysis was performed in triplicate in each of the leaf pools (*B*. *oleracea*-autoclaved and *B*. *oleracea*).

Twelve mg of leaf powder was mixed with 400 μl of 70% (v/v) methanol, preheated to 70 ºC, 10 μl of PbAc (0.3 M) and 120 μl of ultrapure water. Then, 20 μl of glucotropaeolin was added as an internal standard. The tubes were shaken in a microplate incubator (model OVAN Orbital Midi) at 250 rpm for one hour and centrifuged at 3700 rpm for 12 min. Subsequently, 400 µl of the GSL extracts were pipetted onto an ion exchange column with Sephadex DE-AE-A25. By addition of a purified sulphatase solution (E.C. 3.1.6.1, type H-1 from *Helix pomatia*) (Sigma-Aldrich, St. Louis, MO, USA), desulphation was carried out. Finally, the desulphated GSLs were diluted in 200 µl ultrapure water and 200 µl 70% methanol and kept frozen for subsequent analyses.

Chromatographic analyses were performed on an ultra-high performance liquid chromatography system (UHPLC Nexera LC-30AD; Shimadzu, Kyoto, Japan) equipped with a Nexera SIL-30AC injector and a SPDM20A UV/VIS photodiode array detector. The UHPLC column was an X Select ®HSS T3 (2.5 µm particle size, 2.1 × 100 mm i.d.) from Waters Corporation (USA) protected with a Van Guard precolumn. The oven temperature was set at 35 °C. The GSLs were quantified at 229 nm and separated using the following method in aqueous acetonitrile at a flow rate of 0.5 mL min^−1^: 1.5 min at 100% H_2_O, an 11 min gradient from 5 to 25% (v/v) acetonitrile, 1.5 min at 25% (v/v) acetonitrile, a 1 min gradient from 25 to 0% (v/v) acetonitrile, and a final 3 min at 100% H_2_O. Specific GSLs were identified by comparing retention times and UV spectra with standards. GSL standards were purchased from Phytoplan (Diehm and Neuberger GmbH, Heidelberg, Germany). Calibration equations were performed with at least five data points. Specific GSLs were identified by comparing retention times and UV spectra with standards. GSL standards were purchased from Phytoplan (Diehm and Neu-372 berger GmbH, Heidelberg, Germany). Calibration equations were made with at least five data points for the glucosinolates glucoiberin (y = 99397x; R^2^ = 0.950), sinigrin (y = 484871x; R^2^ = 0.994), glucoerucin (y = 276.122x; R^2^ = 0.999), glucobrassicin (y = 869483x; R^2^ = 0.988), and gluconasturtiin (y = 342954x; R^2^ = 0.997).

### Defence hormone analysis

Samples from *R*. *solani*-infected and non-infected bell pepper leaves were collected from 8-week-old plants. For each treatment, leaves were pooled into three pools of three leaves each, collected in liquid nitrogen and stored at − 80 °C. Subsequently, the leaf pools were freeze-dried in a lyophiliser (GAMMA 2–16 LSC plus, Christ, Germany) and mechanically milled to a fine powder in a grinder (Janke and Kunkel A10 mill, IKA-Labortechnik, Staufen, Germany). These samples were used in the hormone and metabolomic analyses.

The main classes of plant defence-related hormones, jasmonic acid (JA), salicylic acid (SA) and ethylene (ET) precursor 1-aminocyclopropane-1-carboxylic acid (ACC) were extracted and analysed as described previously in Albacete et al. (2008), with some modifications. Powdered plant material (0.05 g) was incubated in 1 mL of cold (− 20 °C) extraction mixture of methanol/water (80/20, vol/vol) for 30 min at 4 °C. Solids were separated by centrifugation (20,000 g, 15 min at 4 ºC) and re-extracted for another 30 min at 4 °C with 1 ml of extraction solution. Pooled supernatants were passed through Sep-Pak Plus C18 cartridges (previously conditioned with 3 ml of extraction buffer) to remove interfering lipids and some plant pigments. The supernatant was collected and evaporated at 40 °C with a vacuum. The residue was dissolved in 1 ml methanol/water (20/80, vol/vol) solution using an ultrasonic bath. The dissolved samples were filtered through 13-mm diameter Millex filters with a 0.22-μm pore size nylon membrane (Millipore, Bedford, MA) and placed into opaque microcentrifuge tubes. Ten microlitres of the filtered extract was injected into an Accela Series UHPLC (ThermoFisher Scientific, Waltham, MA) instrument coupled to an Exactive mass spectrometer (ThermoFisher Scientific, Waltham, MA) using a heated electrospray ionisation (HESI) interface. Mass spectra were obtained using Xcalibur software version 2.2 (ThermoFisher Scientific, Waltham, MA). For the quantification of plant hormones, calibration curves were obtained for each analysed component (1, 10, 50 and 100 μg/l) and corrected for 10 μg/l deuterated internal standards. Recovery rates ranged from 92 to 95%.

### Metabolomic analysis

Metabolite extraction and analysis were performed using the methodology previously described by Poveda et al. [15]. Freeze-dried powder (50 mg) was dissolved in 500 mL of 80% aqueous methanol and then sonicated for 15 min. After centrifugation for 10 min (16,000 × *g*, at room temperature), the extract was filtered through a 0.20-µm micropore PTFE membrane and placed in vials for further analysis. For metabolomic composition analysis, we used ultra–performance liquid chromatography (Thermo Dionex Ultimate 3000 LC; Thermo Fisher Scientific, Waltham, MA, USA) coupled to electrospray ionisation time-of-flight mass spectrometry (UPLC-Q-TOF–MS/MS) (Bruker Compact™) with an electrospray ionisation (ESI) source. Chromatographic separation was performed on an Intensity Solo 2 C18 column (2.1 × 100 mm 1.7 µm pore size; Bruker Daltonics, Billerica, MA, USA) using a binary gradient solvent mode consisting of 0.1% formic acid in water (solvent A) and acetonitrile (solvent B). The following gradient was used: 3% B (0–4 min), 3 to 25% B (4–16 min), 25 to 80% B (16–25 min), 80 to 100% B (25–30 min), maintain 100% B for 32 min, 100 to 3% B (32–33 min), and maintain 3% B for 36 min. The injection volume was 5 µL, the flow rate was set at 0.4 mL/min and the column temperature was controlled at 35 °C. MS analysis was performed in the spectrum acquisition range of 50 to 1200 m/z. Both polarities ( ±) of the ESI mode were used under the following specific conditions: gas flow 9 L/min, nebuliser pressure 38 psi, dry gas 9 L/min and dry temperature 220 °C. The capillary and end-plate displacements were set to 4500 and 500 V, respectively. The instrument was externally calibrated with a calibration solution of 1 mM formate/sodium acetate in 50/50 iPrOH/H_2_O with 0.2% formic acid infused directly into the source. Prior to sample injection, the stability of the LC-qTOF system was tested using three consecutive injections of chloramphenicol (ESI mode; ΔRT = 0.02 min; Δm/z = 0.002) and triphenyl phosphate (ESI + mode; ΔRT = 0.02 min; Δm/z = 0.001). Calibration solution was injected at the beginning of each run, and all spectra were calibrated prior to statistical analysis. MS/MS analysis was performed based on the previously determined exact mass and RT and was fragmented using different collision energy ramps to cover a range from 15 to 50 eV. The T-Rex 3D algorithm of MetaboScape 4.0 software (Bruker Daltonics, Billerica, MA, USA) was used for alignment and peak detection.

### Statistical analysis

Statistical analysis of the data was carried out using Statistix 8.0 software. To perform data normality confirmation analysis, the Shapiro–Wilk test was performed. A Student’s t-test was used to compare means at *p* ≤ 0.05 and *p* ≤ 0.01; significant differences were denoted using one or two asterisks, respectively. The group means are represented in columns in the graphs, representing the variance in the form of error bars. In hormone quantification, a one-way analysis of variance (ANOVA) with Tukey’s multiple range test at *p* ≤ 0.05 was used for pairwise comparisons.

Statistical analysis of the metabolomic data was performed using web-based software Metaboanalyst [33]. To remove uninformative variables, the data were filtered using an interquartile rank filter (IQR). In addition, Pareto variance scaling was used to remove variances and adjust the importance of high and low abundance ions to an equal level. The resulting three-dimensional matrix (peak indices, samples and variables) was further subjected to statistical analysis. Partial least squares discriminant analysis (PLS-DA) was constructed to determine the metabolic differences between treatments. PLS-DA models were cross-validated using the R2 and Q2 parameters. The quality assessment (Q2) and R-squared (R2) statistics provide a quantitative measure of consistency between the predicted and original data or, in other words, estimates the predictive ability of the model. The PLS-DA model, using the first principal component of variable importance in the projection (VIP) values, was used to find differentially expressed metabolites. Based on VIP > 2, metabolites related to resistance were distinguished.

### Tentative metabolite identification

For tentative identification, a consensus molecular formula was assigned to each molecular feature based on exact mass data and isotopic pattern distributions for the precursor using MetaboScape 4.0 and Sirius v4 [34] software. A molecular formula was used to perform identification analysis on publicly available databases: PubChem [35], MassBank [36], Kyoto Encyclopedia of Genes and Genomes (KEGG) [37], KNApSAcK [38], Metlin [39] and Chemspider [40]. When available, the ms/ms fragmentation spectrum of the reference compounds identified in the databases was compared to that obtained experimentally.

## Results

**Fig. 1:**
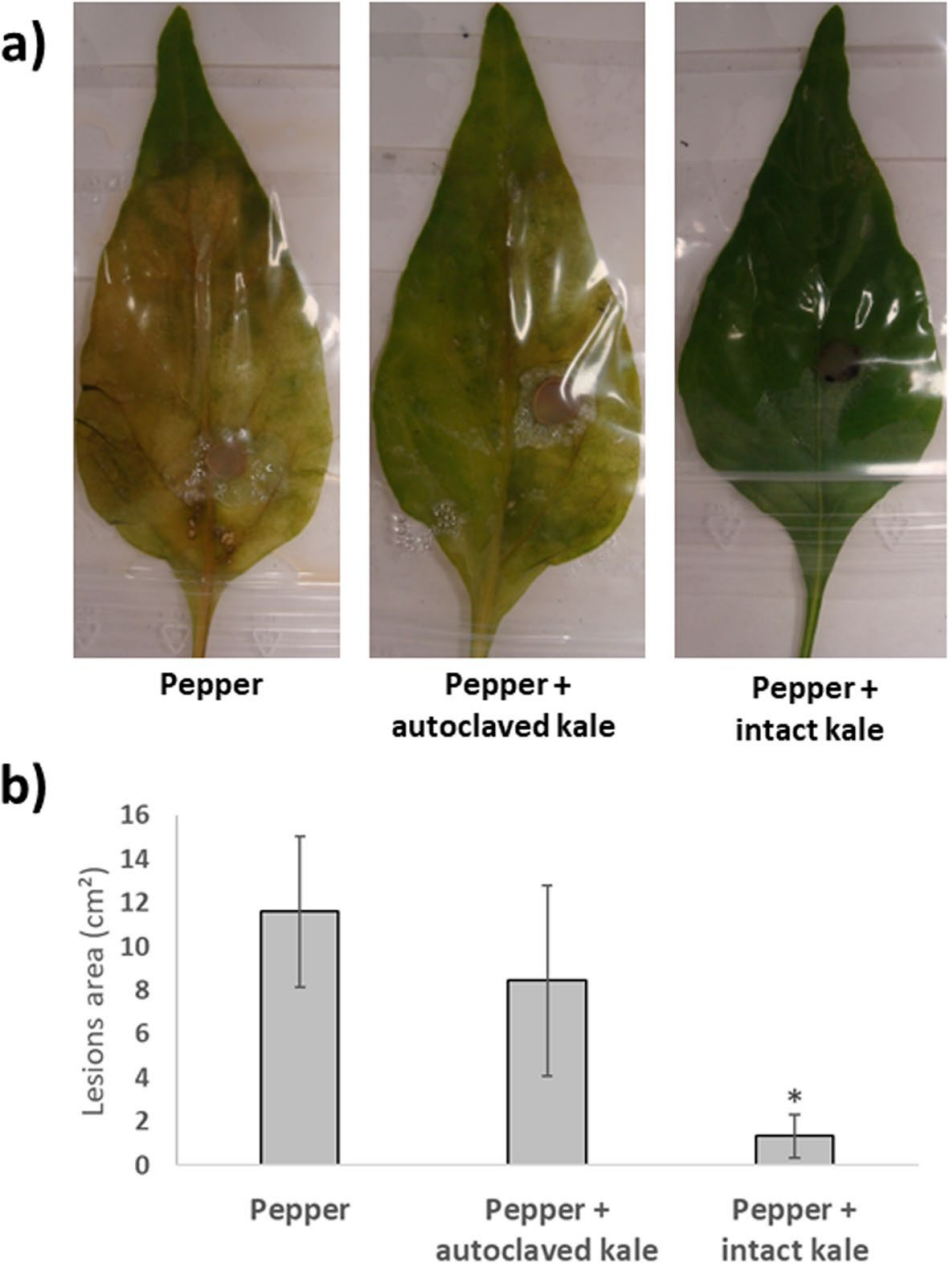
*Rhizoctonia solani*-infected pepper leaves (**a**) and lesion area quantification (cm.^2^) **b**. Pepper plants without kale GrM (Pepper), with autoclaved kale GrM (Pepper + autoclaved kale) or with kale GrM (Pepper + intact kale). Data are the mean of three groups of 10 leaves for each condition with the corresponding SEM. Student’s *t*-test was performed. Asterisks denote significant differences at *p* ≤ 0.05 (*)

To determine the possible capacity of inducing SAR against *R*. *solani* in bell pepper plants using kale GrM, we added FD-GrM and AUT-GrM to the pepper substrate. Leaves of control plants inoculated with *R*. *solani* showed chlorosis and brown spots due to fungal development (Fig. 1a). A similar performance was observed in pepper plants treated with AUT-GrM. However, the leaves of pepper plants treated with FD-GrM barely showed chlorosis or brown spots. These observations were corroborated by measuring the area of the lesions (Fig. 1b). No significant differences were observed in *R*. *solani* leaf injury between plants treated with AUT-GrM and non-inoculated control plants, whereas plants inoculated with FD-GrM showed significantly smaller lesions than the control and *B*. *oleracea*-autoclaved plants (a reduction of 88% of the lesion area compared to the control and 85% compared to plants treated with AUT-GrM).

### Pepper leaf defence hormones

To determine how FD-GrM treatment may influence phytohormone production in pepper plants, we analysed 12 hormones (Table 1). Phytohomones can be classified as defensive (SA, JA, ABA or ET) or growth-related hormones (auxins, gibberellins and cytokinins). In the former group, we observed an impact of treatment with FD-GrM on the biosynthesis pathways of SA and ET in pepper plants. The content of SA was similar in control plants and in FD-GrM-treated plants, but after inoculation with *R*. *solani*, the level of SA significantly decreased in control plants but increased four-fold in FD-GrM-treated plants, from 747 to 2944 ng/g. Prohormone ACC is a direct precursor of ET. Basal levels of this hormone were lower in FD-GrM-treated plants than in control plants. When inoculated with *R*. *solani*, the control plants did not modify the level of ACC, but in FD-GrM plants, ACC increased almost 300%. No significant differences were observed in JA or ABA biosynthesis among treatments.

**Table 1:** Mean concentration (ng/g) of the different hormones analyzed in pepper leaves

						**Cytokinins**			**Gibberellins**			**Auxin**	
**Substrate**	**RS Inoculation**	**ACC**	**SA**	**ABA**	**JA**	**tZ**	**ZR**	**iP**	**GA3**	**GA1**	**GA4**	**IAA**	**Mel**
Soil	Yes	926.3bc	379.0bc	195.7	586.6	0.0b	2790.2ab	815.6	83.1a	27.7	50.9	13.3bc	18.5
Soil	No	1151.3b	1038.0b	203.6	667.9	1.7b	3432.9a	1762.4	49.9ab	3.2	6.3	29.7b	18.3
FD-GrM Kale	Yes	1679.2a	2944.1a	178.9	646.6	15.7a	784.4bc	1209.7	25.0b	0.2	7.7	63.7a	0.0
FD-GrM Kale	No	654.1c	747.7c	203.0	579.9	0.0b	447.2c	1386.2	50.5ab	5.5	8.7	5.0c	0.0

In each column, means with different letters indicate significant differences at probability ≤ 0.05

**Fig. 2:**
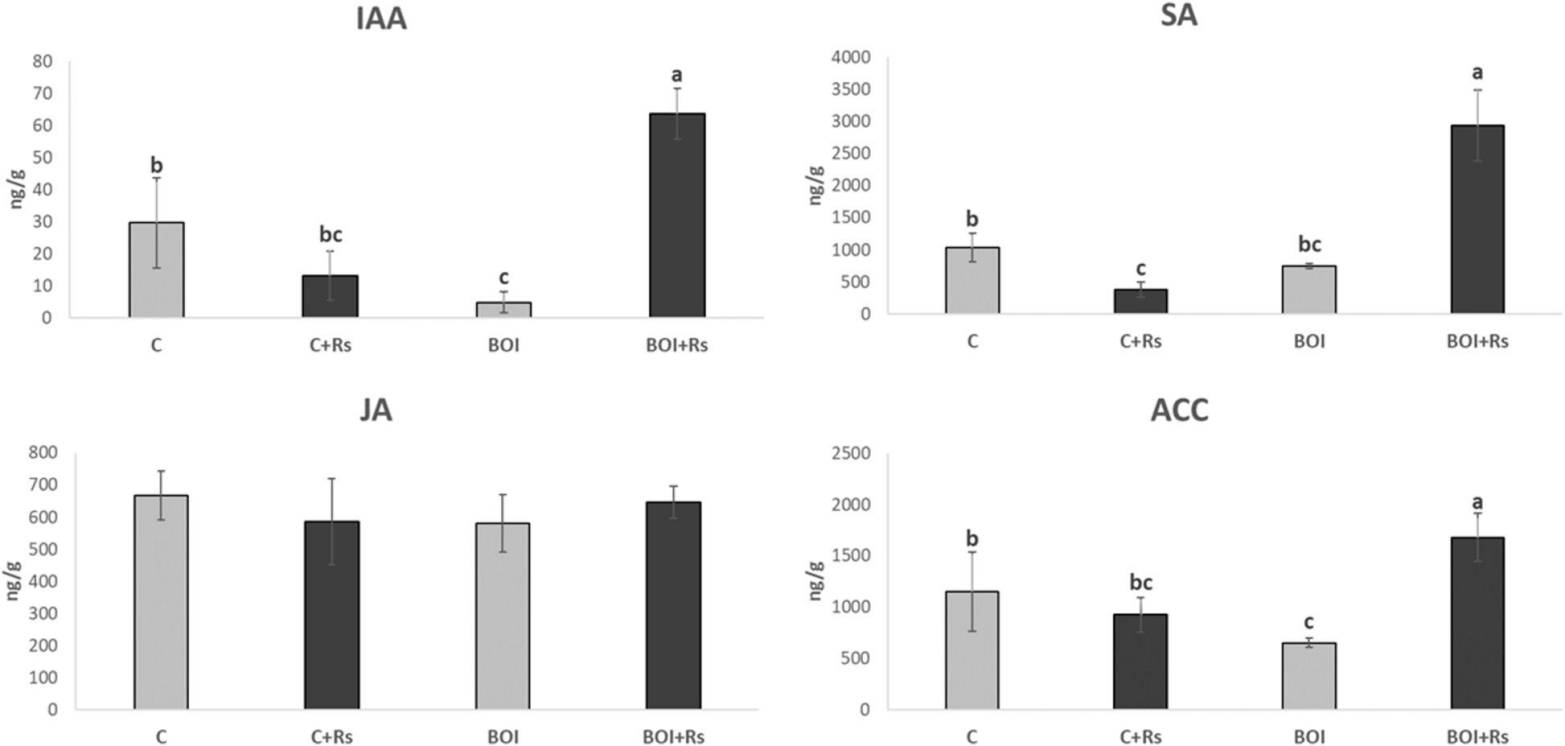
Defence hormone content in leaves of pepper plants root-inoculated with kale GrM (***B***. *oleracea*) or without kale GrM root application (**C**) and infected with *R*. *solani* (+ Rs). SA: salicylic acid, JA: jasmonic acid, ACC: 1-aminocyclopropane-1-carboxylic acid. Data are the mean of three pools of three pepper leaves with the corresponding SEM. One-way analysis of variance (ANOVA) was performed, followed by Tukey’s test. Different letters represent significant differences (*p* ≤ 0.05)

Hormones that regulate plant growth are associated with trade-offs between growth and defence. For this reason, we also studied the profile of these hormones in pepper plants. Gibberellins did not show any significant differences among treatments. Within cytokinins, a low quantity of tZ was present in control plants and nothing in FD-GrM-treated plants. However, when inoculated, the level of tZ disappeared in the control but increased to 15 ng/g in FD-GrM-treated plants. Regarding auxins, IAA also showed a lower quantity in FD-GrM-treated plants compared to control plants, but it was highly stimulated in FD-GrM plants when inoculated with *R*. *solani* (Fig. 2).

### Metabolomic profile of pepper leaves treated with lyophilised kale GrM

**Fig. 3:**
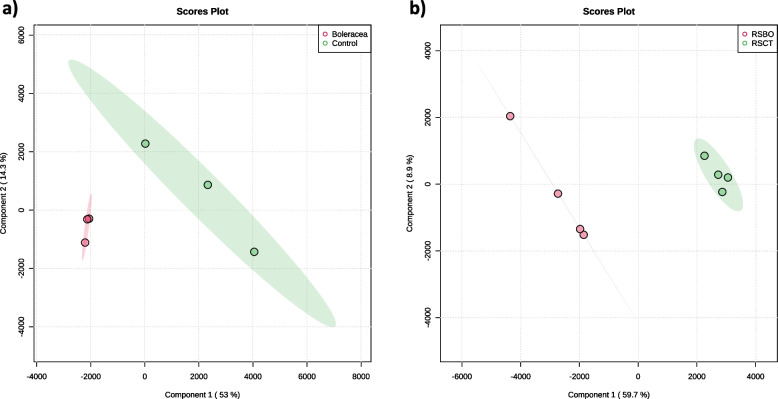
PCA score plots to pepper leaves root-inoculated with kale GrM (*B*. *oleracea*) or without kale GrM root application (**C**) without *R*. *solani* infection (**a**) and with *R*. *solani* infection (**b**)

**Fig. 4:**
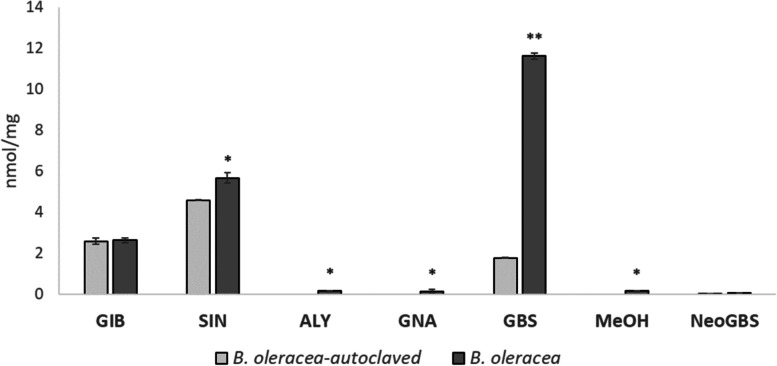
GSL content in leaf pools used as GrM from kale (*B*. *oleracea*) or autoclaved kale (*B*. *oleracea*-autoclaved). GIB: glucoiberin, SIN: sinigrin, ALY: glucoalyssin, GNA: gluconapin, GBS: glucobrassicin, MeOH: metoxy-glucobrassicin, NeoGBS: neoglucobrassicin. Data are the mean of three technical replicates with the corresponding standard error of the mean (SEM). Student’s t-test was performed between each GrM for each GSL. Asterisks denote significant differences at *p* ≤ 0.05 (*) and *p* ≤ 0.01 (**)

To explore the metabolomic changes that take place on pepper leaves induced by FD-GrM, which can protect the plant against *R*. *solani*, we performed an untargeted metabolomic analysis. Partial least squares discriminant analysis (PLS-DA) was carried out to investigate and visualise the patterns of metabolite changes between the infected and non-infected plants. In the first study, non-infected plants growing with FD-GrM or on soil (control) were used to detect those metabolites affected by FD-GrM treatment (Fig. 3a). In the second study, the same comparison was made for *R*. *solani*-infected plants (Fig. 3b). A PLS-DA plot was generated for both treatments (Fig. 3). The PLS-DA model was evaluated through cross-validation (R^2^ and Q^2^ parameters). The quality assessment (Q^2^) and R^2^ statistics provide a qualitative measure of consistency between the predicted and original data or, in other words, estimated the predictive ability of the model. For comparison, features with a VIP score > 2 in the PLS-DA model were selected and considered the most influential features. Finally, by comparing both datasets, we selected the 26 features that were only present in the comparison between *R*. *solani*-infected plants with and without FD-GrM application. We tentatively assigned compound names to 16 of 26 metabolites (Table 2). The compounds were identified as phenolics (19%), mainly flavonoids (malonylapiin, cyanidin 3-[6''-malonylsambubioside]) and coumarins (hydroxycoumarin), carboxylic acid (pipecolic acid, gluconic acid) (12%), amine oxides (dodecyldimethylamine oxide) (6%), glycerols (monolinolenin) (19%), sterioids (polypodine B) (6%), and lysophospholipids (19%). Interestingly, we identified glucobrassicin (GBS) and glucosinolate fragments (GSLs) in pepper tissues. Glucosinolates are secondary metabolites that appear almost exclusively in plants of the Brassicaceae family. We detected GBS only in pepper plants treated with FD-GrM when infected with *R*. *solani*. Lyophilisation is a soft process that conserves the metabolomic profile of the tissue, whereas autoclaving results in metabolite degradation. To confirm the degradation of GSLs in autoclaved tissues, we performed a target metabolomic analysis. Sinigrin (SIN), glucoalyssin (ALY), gluconapin (GNA) and methoxy-glucobrassicin (MeOH) content decreased significantly (by 19, 18, 13 and 16%, respectively), and the glucobrassicin (GBS) content significantly decreased even more (by 85%), compared to levels observed in kale leaf tissues (*B*. *oleracea*). There were no significant differences in glucoiberin (GIB) or neoglucobrassicin (NeoGBS) content between AUT-GrM and the control (Fig. 4).

**Table 2:** Tentative identification of metabolites selected after a PLS-DA analysis, responsible of the resistance to RS in pepper

RT (s)	Neutral mass	mz	Formula	Tentative name	msms
536	129.05807	130.065	C9H7N	Fragment of Desulfoglucobrasscin	51.025, 86.101, 97.008, 105.044, 128.048
61	129.07874	130.086	C6H11NO2	Pipecolic acid	56.05, 84.081
61	135.05613	136.0634	C4H9NO4		65.04, 68.997, 79.043, 94.067, 110.061, 119.035
59	137.04759	138.0548	C4H8FNO3		51.024, 65.04, 94.067, 105.045, 110.061, 136.065
530	162.03226	163.0395	C9H6O3	Hydroxycoumarin	53.099, 63.023, 89.039, 105.046, 117.035, 135.044
57	196.0580	195.0508	C6H12O7	Gluconic acid	
57	243.0588	242.0516	C10H13NO4S		
535	448.0610	447.0537	C16H20N2O9S2	Glucobrassicin	74.990, 96.957, 259.011
1536	451.31547	452.3227	C22H46NO8	PC(O-14:1(1E)/0:0)	
52	121.91728	122.9245			
61	141.11497	142.1222	C8H15NO		
1337	229.24055	230.2478	C14H31NO	dodecyldimethylamine oxide	45.058, 53.04, 58.066, 62.062
1475	260.21404	261.2215	C18H28O	(E)-octadec-13-en-9,11-diyn-1-ol	67.054, 79.055, 91.055, 121.101, 135.117, 243.209
52	289.83974	290.847			94.93, 122.925, 204.87, 247.912
1779	312.15510	313.1624	C14H26O6		
1475	352.26186	353.2651	C21H36O4	1-Monolinolenin	67.055, 81.07, 135.117, 243.211, 261.221
1484	453.28690	454.2938	C21H44NO7P	1–16:0-lysoPE (1-palmitoyl-sn-glycero-3-phosphoethanolamine)	62.061, 282.28, 313.273, 339.24, 436.283
1453	477.28644	478.2937	C23H44NO7P	1–18:2-lysoPE (1-(linoleoyl)-sn-glycero-3-phosphoethanolamine)	62.061, 216.064, 306.282, 337.273, 460.282
61	483.20563	162.0758	C23H29N7O3S		51.024, 53.039, 82.066, 100.076, 105.046
1475	496.30415	497.3123	C27H44O8	Polypodine B	127.04, 183.065, 261.222, 335.258, 405.263, 479.3
1475	514.31518	515.3224	C27H46O9		
1416	517.31793	518.3252	C26H48NO7P	LysoPC 18:3	86.097, 104.107, 124.999, 184.073, 500.308
1510	521.34918	522.3564	C26H52NO7P	LysoPC 18:1	86.097, 104.107, 124.999, 184.073, 504.343
1475	560.31916	559.3119	C28H48O11		
1124	650.15018	651.1575	C29H30O17	Malonylapiin	57.035, 97.029, 115.04, 127.039, 271.06, 519.114
1060	666.14474	667.152	C29H30O18	Cyanidin 3-(6''-malonylsambubioside)	69.034, 97.029, 287.055, 535.109, 581.152

## Discussion

The problems associated with the agricultural use of synthetic chemical fungicides increase the need to find new alternatives to combat fungal diseases in crops [2–4]. Different GrMs have been widely used in the control of soil-borne pathogens directly through the presence of antimicrobial phytochemicals or by modifying soil physicochemical and microbial conditions. However, the use of GrM as a potential elicitor resource capable of activating SAR in plants has not been addressed [41]. Plant tissues can function as elicitors of a plant defensive response, as is the case with small cell wall fragments released after a pest or pathogen attack, the so-called damage-associated molecular patterns (DAMPs) [42]. In this sense, numerous molecules and metabolites of plant origin capable of functioning as elicitors in crops, such as polysaccharides [43], secondary metabolites (including allicin, naringin and terpenes) [44], and plant defence hormones and derivates, have been described [45].

In our work, the application of FD-GrM from kale leaves caused the activation of an SAR against the foliar attack of *R*. *solani* in pepper plants, which did not occur with AUT-GrM. The use of FD-GrM did not have a major impact on the systemic content of any of the defence hormones in pepper plants, indicating that this strategy does not work for the activation of systemic defences in the absence of a pathogen. However, in the presence of the pathogen, kale FD-GrM application led to a systemic increase in SA and ACC (ET precursor) content, indicating that both hormonal pathways were involved in the observed SAR. These results suggest that FD-GrM act by priming defence mechanism in the absence of the pathogen. Although it is generally established that the SA pathway acts against biotrophic pathogens and the JA/ET pathway acts against necrotrophs, there is increasing evidence of a continuous antagonistic and synergistic interaction of both hormone defence pathways against each pathogen [46]. In our work, we showed how both SA and ET seem to be involved in the SAR obtained against *R*. *solani* using kale FD-GrM.

SAR in pepper plants against *R*. *solani* induced by FD-GrM was related to an increase in the leaf-specific content of several secondary metabolites. Among them, hydroxycoumarin [47], gluconic acid [48], GBS [49] or cyanidin [50] stand out for their antifungal activity. Other identified secondary metabolites have other functions, such as pipecolic acid, which is involved in amplifying SA-mediated plant immunity [51]. In addition, we identified the main GSLs, GBS and its derivative desulpho-GBS, from kale manure in pepper leaves. Pepper plants do not synthesise GSLs, and when plants grow on kale manure, GBS is not present in pepper leaves. However, after inoculation with *R*. *solani*, the pepper leaves accumulated GBS. To confirm that autoclaved tissue affects the content of GSLs in kale tissue, we performed a targeted metabolomics analysis. The results indicate a significant reduction in the main glucosinolate GBS (85%) and a reduction in other minority glucosinolates, SIN (19%), ALY (18%), MeOH (16%) and GNA (13%). It has been previously described how, at temperatures above 100 °C, all GSLs present in cabbage (*B*. *oleracea* var. *capitata*) are degraded, with GBS and its derivatives being the first GSLs to be degraded [52]. In this regard, the water content of *Brassica* tissues is critical for the thermal degradation of GSLs. In broccoli (*B*. *oleracea* var. *italica*), total degradation of GSLs is achieved with water content of 82% and temperatures up to 100 °C. However, with a water content of 13% in tissues, GSL degradation does not occur until above 120 °C [53]. Therefore, the use of AUT-GrM is a good strategy for removing GSLs from kale tissues. This finding is an important starting point for future work to explain why this happens. Thus far, we can only hypothesise that foliar attacks on pepper plants cause them to take up secondary metabolites through the roots, such as GBS, mobilising them through the xylem to the aerial part of the plant. In this regard, the antifungal capacity of GBS has been extensively described against pathogens, such as *Alternaria brassicae* and *Sclerotinia* *scletoriorum* [21]. Nevertheless, it is necessary to consider that the roots of pepper plants can absorb other metabolites; thus, more research is needed to confirm the effect of GBS on pepper resistance.

In conclusion, FD-GrM can activate systemic defensive responses in pepper plants against foliar infection by the necrotrophic pathogen *R*. *solani*. This plant response could be due to the action of several metabolites present in kale tissues. Between them, GBS and GSLs from *Brassica* tissues, not naturally synthesised by pepper plants, could have a role as elicitors. The hormonal pathways involved in this SAR involve SA and ET, leading to foliar accumulation of different antifungal compounds against *R*. *solani*, such as hydroxycoumarin or gluconic acid.

## Data Availability

The datasets used and analysed during the current study are available from the corresponding author upon reasonable request.
